# Enhanced Evidence-Based Chinese Medicine Clinical Practice Guidelines in Hong Kong: A Study Protocol for Three Common Diseases

**DOI:** 10.1155/2015/482706

**Published:** 2015-03-01

**Authors:** Nannan Shi, Linda L. D. Zhong, XueJie Han, Tat Chi Ziea, Bacon Ng, Zhaoxiang Bian, Aiping Lu

**Affiliations:** ^1^Institute of Basic Research in Clinical Medicine, China Academy of Chinese Medical Sciences, Beijing 100700, China; ^2^School of Chinese Medicine, Hong Kong Baptist University, Hong Kong Special Administrative Region, Hong Kong; ^3^Chinese Medicine Department, Hong Kong Hospital Authority, Hong Kong

## Abstract

We presented a study protocol of developing Chinese medicine clinical practice guidelines for three common diseases in Hong Kong, including insomnia, chronic gastritis, and cerebral infarction. This research project will be conducted in three phases. First phase is the preparation stage which consists of the establishment of steering committee and panel. Second phase involves 6 steps, which are searching and identifying evidence, text mining process, Delphi survey, synthesizing of data, consensus conference, and drafting guidelines. In this phase, text mining technique, evidence-based method, and formal consensus method are combined to get consolidated supporting data as the source of CM CPGs. The final phase comprised external reviews, dissemination, and updating. The outputs from this project will provide three CM CPGs for insomnia, chronic gastritis, and cerebral infarction for Hong Kong local use.

## 1. Background

Clinical practice guidelines (CPGs) are commonly defined as “systematically developed statements to assist practitioner and patient decisions about appropriate health care for specific clinical circumstances” [1]. They can improve health outcomes, the quality of clinical decisions, efficiency, and optimistic value for money [2]. Several major medical organizations, including the American Medical Association (AMA) and the Institute of Medicine (IOM), have carefully formulated methodology for developing scientifically sound guidelines. In Hong Kong, Hong Kong Academy of Medicine also developed local CPGs on the basis of the consensus of experts and the guidelines formulated by the major medical organizations worldwide mentioned above [3].

Chinese medicine (CM) is a traditional medical system that originated in China on the basis of clinical experience and theory, and it has, to a varying degree, been disseminated to and used in many countries. The increased use of CM has attracted public concern over the issue of its secure application. In the past few years, problems concerning the side effects induced by the incorrect use of CM have arisen (e.g., renal toxicity caused by long-term use of Manchurian Dutchman's pipe or Fang chi root [4, 5]). To ensure the safety and quality of CM practice, Chinese medicine clinical practice guidelines (CM CPGs) have been developed [6, 7]. In China, the early CM CPGs have emerged since the 1980s. Until now there are almost 500 CM CPGs written by different professional organizations in China according to routine press briefing of State Administration of Traditional Chinese Medicine of China (SACM) in December 2013. To provide more trustworthy CM CPGs, China Academy of Chinese Medical Sciences had developed evidence-based CM CPGs [8], which were the first evidence-based CM CPGs in the world through international multidisciplinary collaboration with more than 200 experts.

In Hong Kong, there are no CM CPGs published by any authority currently to guide CM clinical practice. Most of the clinical practices of CM practitioners are still based on the individual experiences and lack the strong evidence to support the treatment scheme. The differences between CPGs in Hong Kong and mainland China would be the evidences involved or bilingual published language. With the increasing number of patients seeking CM services, there is an urgent need to develop CM CPGs to standardize CM practice and promote evidence-based CM practice. In 2013, Hospital Authority in Hong Kong had launched the project of development of CM CPGs. In this study protocol we aim to develop CM CPGs in three areas which are of high disease burden and the evidence of effective practice is available in Hong Kong. The three areas include insomnia, cerebral infarction, and chronic gastritis.

## 2. Methods/Design

**Figure 1:**
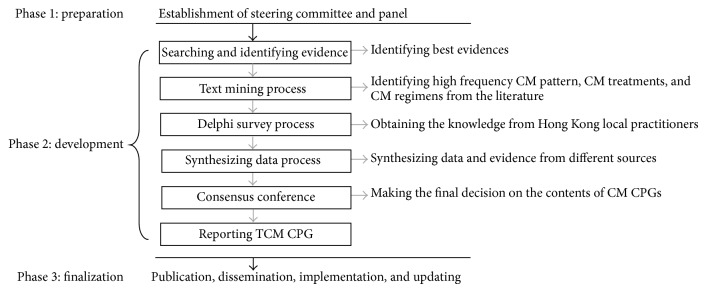
Flowchart of development process of CM CPGs.

The methods will follow the general CPG development process [9–17] with modifications for achieving solid data. The research program will be undertaken in 3 phases, including preparation phase, development phase, and finalization phase (Figure 1).

### 2.1. Phase I: Preparation

A steering committee is ultimately responsible for the development of CM CPGs for three common diseases in Hong Kong. They control the quality of whole development process and determine the contents of CM CPGs. A panel is established as technical assistant team to prepare related guideline documents, review the literatures, conduct Delphi survey, organize the consensus conference, and draft and edit the CM CPGs.

### 2.2. Phase II: Development

In this phase, drafting CM CPGs will be completed. First, for providing adequate and consolidated supporting data, we will collect basic information as “potential content item” derived from the literature evidence, text mining process, and Delphi survey. Second, we will synthesize “potential content item” after evaluating its supporting data. Third, consensus conference will be held to make final decision on whether those “potential content items” can be involved in the CM CPGs.

### 2.3. Searching and Identifying Evidence

The aim of this step is to identify the best available evidence to address the related topics in three CM CPGs.

#### 2.3.1. Literature Searching

A systematic search of clinical research regarding CM treatments for insomnia, cerebral infarction, and chronic gastritis will be conducted. All relevant published studies in Chinese or English will be identified by searching the following databases: all EBM reviews, including Cochrane DSR, ACP journal Club, DARE, CCTR, CMR, HTA, And NHSEED; EMBASE (1980–2013), EMBASE Classic (1947–1979); Ovid MEDLINE(R) (1950–2013); Ovid OLDMEDLINE(R) 1948–1965; China Journals Full-text Database (1994–2013); CNKI (1979–2013). The following search terms will be used:

1. “chronic gastritis” AND “Chinese herb” OR “acupuncture” OR “massage” OR “Chinese medicine” OR “Traditional Chinese Medicine”;
2. “insomnia” AND “Chinese herb” OR “acupuncture” OR “massage” OR “Chinese medicine” OR “Traditional Chinese Medicine”;
3. “cerebral infarction” AND “Chinese herb” OR “acupuncture” OR “massage” OR “Chinese medicine” OR “Traditional Chinese Medicine.”

#### 2.3.2. Eligibility Criteria

Two reviewers will independently search the journal and select potentially relevant articles after screening the titles and abstracts. In case of uncertain eligibility, the full text will be screened. Eligibility criteria have been set as follows.

1. Types of studies: we will include published CPG, systematic review, meta-analysis, and original clinical researches including randomized clinical trials (RCT), cohort study, case series, and case report on treatments and prevention of insomnia, cerebral infarction, and chronic gastritis with CM approaches.
2. In original clinical research, considering interventions in the treatment group, the following interventions will be included:
  - Chinese herb medicine: single herb or herb formula,
  - acupuncture and moxibustion: general acupuncture therapy, electroacupuncture, and moxibustion,
  - other CM treatments: external application of the traditional Chinese medicine, acupoints application therapy, and CM regimen.
3. We will exclude trials or studies reported as “animal studies,” “editorials,” or “others”; use the treatment as extracts from a single herb, Chinese proprietary medicine irrespective of preparation (e.g., oral liquid, tablet, capsule, pill, powder, plaster, or injection liquid) with mode of delivery (e.g., oral, cutaneous, intramuscular, or intravenous injection).

#### 2.3.3. Evaluation of Evidence

Once the articles are selected as potential sources of evidence, the methodology used in each study will be assessed to ensure its validity. Two researchers will review the literature independently. For assessing the methodological quality of different types of study, we will use different tools as follows: AMS TAR [18] tool for SR or meta-analysis, Jadad scale [19] for RCT, STROBE checklist for observational studies, and CARE [20] checklist for case studies.

#### 2.3.4. Level of Evidence

Considering the characteristics of CM research, we utilize the well-accepted evidence grading systems for CM [21] established by Liu. And the evidence body has been successfully used in the development of first evidence-based CM CPGs [8].

#### 2.3.5. Dealing with Existing CM CPGs

If existing CM CPGs have been identified, we will use AGREE II [22] for evaluating methodological quality. Then the eligible CM CPGs will be submitted to steering committee for further evaluation. Steering committee will review integrated contents of existing CM CPGs including scope, target patients, and CM interventions to evaluate the possibility of local adaptation. Steering committee will decide whether the existing CM CPGs should be adapted.

### 2.4. Text Mining Process

The purpose of text mining process is to provide more information and data for developing CM CPGs, especially in those areas lacking high quality evidences, like classification of CM patterns. High frequency CM patterns and related CM treatments including Chinese herb treatments, acupuncture, and moxibustion will be obtained from the literature [23].

#### 2.4.1. Process Design

High frequency CM patterns and related CM treatments reported in clinical studies imply that they are well known by practitioners to some extent. We consider this high frequency information as a kind of supporting data for forming CM CPGs. We will use a data slicing algorithm based on the calculation of frequency, which is detailed in our previous research [24], to filter high frequency CM pattern and related CM treatments.

#### 2.4.2. Source of Data

To avoid repetition of clinical study and ensure efficiency, we will choose only one database as data source. We will identify clinical researches with CM treatment for chronic gastritis and insomnia and cerebral infraction in CNKI (http://www.cnki.net/), which is the most comprehensive electronic medicine database in China. The time range is from 1979 to January 2014. The following search terms will be used: “chronic gastritis” OR “insomnia” OR “cerebral infarction” (in Chinese). The identified studies will be downloaded from CNKI and its plain TXT data will be transferred into database (Microsoft SQL 2000) for further use.

### 2.5. Delphi Survey Process for Local Practitioners

The aim of Delphi survey is to obtain the knowledge from Hong Kong local practitioners. The Delphi process will comprise a series of semistructured questionnaire and replicate survey rounds until consensus is achieved. We will separately conduct Delphi survey for 3 different clinic topics, including CM diagnosis and therapy for insomnia, chronic gastritis, and cerebral infraction.

#### 2.5.1. Forming Questionnaire

Participants will be informed by the evidence and information derived from text mining process for leading an effective process. All of the identified evidences and information will be listed in the initial questionnaire. Items will be grouped under the following broad headings: (1) classification of CM patterns; (2) Chinese herb treatments; (3) acupuncture and moxibustion treatments; and (4) CM regimen. Any response provided by participants in the first round survey that is not included in existing evidence and information will be added and returned to the participants in the subsequent rounds, along with a new questionnaire to answer. The questionnaires will be developed and delivered by an online questionnaire platform, which is Qualtrics Survey Software.

#### 2.5.2. Participants

We will invite local CM practitioners via email which will outline the aim and process, likely time commitment, and process of the Delphi survey. Local CM practitioners with more than 5 years of clinical experience in Hong Kong will be randomly selected from Hong Kong Registered Chinese Medicine Practitioners Association, which is the biggest professional organization of CM in Hong Kong. It is proposed to involve 90 participants for Delphi survey focused on three different topics. A reminder will be sent to those selected experts who do not respond by the deadline given. Nonrespondents to this reminder are considered to be uninterested in the study and will not be contacted again. All participants will be allocated a random identification number for reporting and collation of the results. Demographic data regarding occupation/field, place of employment, education background, degree, and years of professional experience will be recorded.

#### 2.5.3. Criteria of Consensus

There are no currently accepted criterion standards to determine whether a consensus has been reached. For the purpose of this study consensus will be deemed achievable for each item of >80 percent agreement indicating substantial to excellent agreement. Any items that reach consensus will not be required for further comment and will be included in a separate page of the survey.

#### 2.5.4. Ethics

Ethical approval has been obtained from Hong Kong Baptist University Research Ethics Committee.

### 2.6. Synthesizing of Data

The aim of this step is to synthesize the data and evidence from different sources. The data and evidence generated from literature evidence, text mining process, and Delphi survey will be considered as “potential content items” after evaluating and synthesizing.

#### 2.6.1. Rating “Potential Content Items”

All of the “potential content items” will be rated from 1 to 3 points. A “potential content item” will score 1 point if it is supported by one source of data. As there are three sources of data, the maxima and minima points will be 3 to 1. The “potential content items” with the situations below will score 1 point:

1. “potential content item” is supported by any of the literature evidences regardless of whether level of evidence will score 1 point;
2. “potential content item” is identified by text mining as high frequency item will score 1 point;
3. “potential content item” reaching consensus in Delphi survey will score 1 point.

#### 2.6.2. Synthesizing “Potential Content Items”

We will classify and synthesize “potential content items” according to their achieved scores. All of the “potential content items” will be classified as below.

1. If “potential content item” is rated as 1 point, it will not go to the next step. A “potential content item” has high-level supporting data, like high-level evidence, high consensus, and high frequency, but only achieves support from one kind of source. It will be marked as parking lot item which had been separately recorded and reported to consensus conference for further consensus.
2. If “potential content item” is rated >1 point, it will be submitted to the consensus meeting.

All of the “potential content items” will be grouped and listed under the following synthesizing tables (Tables 1, 2, and 3) for easy collection and synthesizing of data: (1) classification of CM patterns; (2) Chinese herb treatments; (3) acupuncture and moxibustion treatments; and (4) CM regimen.

### 2.7. Consensus Conference

The purpose of this step is to make the final decision on the contents of CM CPGs. In this step, all “potential content items” with supporting data will be reviewed and consensus will be achieved on “potential content item” whether to be included in the new guidelines.

#### 2.7.1. Process Design

A one-day consensus development conference will be held to determine guidelines' content, rather than wording or format. At the first half day, the results of synthesizing process will be provided to participants, and the conference will include background presentations to ground conversations on empirical information to facilitate cohesive discussion. Participants will be led in structured discussions of each potential item generated from the synthesizing process. Care will be taken to ensure that all participants express views, that all ideas are discussed in depth, and that assertive participants do not dominate the discussion. The observers will record the points from the participants and decisions generated by conference and draft resolution. At the second half day, the resolution will be circulated to all the participants in consensus group. Revisions will be made on resolution based on these responses. The process will replicate until consensus is reached. Every participant will be required to sign on the resolution if the resolution accurately represented the decisions made during the meeting.

#### 2.7.2. Participants

Participants will include all members of steering committee, and one independent observer. Panel will act as assistant in consensus conference.

### 2.8. Drafting Guidelines

Reporting CPG in right way can promote the understanding of CPG and advance a transparent CPG's development process [25]. For this reason, it is necessary to develop a standardized reporting guideline for CM CPG. We have developed a reporting checklist for CM CPG including 10 topics and 19 subitems according to the existing reporting guideline. The final guideline will be reported following the reporting checklist (Table 4).

### 2.9. Phase III: Finalization

In this stage, external review, dissemination, implementation, and further updating of CM CPG will be completed.

#### 2.9.1. External Reviews

External review process will be conducted once a CM CPG has been drafted to ensure its methodological quality and being representative of the comprehensive perspectives of related professionals. We will invite 3 to 5 methodologists to review the CM CPGs particularly focusing on the methodology quality of the development process. Agree II will be used as evaluation tool for methodology quality.

#### 2.9.2. Dissemination

The full content of guidelines will be linked on the website of professional organization and open to public. Simultaneous publications in multiple, peer-reviewed journals will begin the process of dissemination and uptake.

#### 2.9.3. Updating

In addition, it is widely believed that CPG rapidly becomes outdated and therefore requires updating in time [22]. Steering committee will ask for collecting information of latest research and signal the need for updating as new evidence or consensus arises. Emerging evidence or consensus will be reviewed by all members of GDG to consider whether it can potentially affect current clinical management strategies. Once new evidence or consensus necessitates changing contents of CM CPG, revised version of CM CPG will be completed and published.

## 3. Discussion

These methods described above are applied to develop the trustworthy CM CPGs based on consolidated data for Hong Kong local use. Because of the limitations existing in traditional methods of development of CM CPG, some improvements are made in our research for strengthening the supporting data. To address the reasons for improvements, we analyze the defects of traditional methods used in developing CM CPGs.

In general, there are 3 kinds of traditional methods for developing CPGs, which are consensus development method, evidence-based method, and evidence-consensus based method. All of the 3 methods are difficult individually to develop the trustworthy CM CPGs based on consolidated data. When using evidence-based method to develop CM CPGs, the lack of high quality clinical evidence becomes the key barrier. Inevitable and inherent defects existing in the design of CM clinical research make it more difficult to achieve high-level evidence. For example, it is hard to design good controlled CM RCTs with highly individualized therapeutic schemes and vague diagnosis criteria. When using consensus development method, real consensus is hard to be obtained in the process of development of CM CPGs. On the one hand, a vast majority of consensus-based CM CPGs are developed by informal group. Such informal group sometimes terms simple consensus group which originates from the same research team to discuss a problem with the aim of research agreement. On the other hand, the basis of consultation in the consensus process is commonly generated from expert opinions or unsystematic literature reviews that result in bias by individual views and lack of objective data leading to misunderstanding in consensus process. The consensus generated from such kind of informal consensus method can hardly represent the real opinions of all the experts. Otherwise, although evidence-consensus based method provides a possibility of developing more informative and reliable CM CPG, the so-called “evidence-consensus based method” in CM CPG is simple merge of existing methods. It lacks clear procedure, necessary criteria for combination data from evidence and expert opinion; to some extent the limitations of both methods are unavoidable in the new method for developing CM CPG.

Based on these concerns, there are some improvements in our development procedure of CM CPGs.

*Firstly*, we chose mixed methods rather than one method to develop CM CPGs for achieving more supporting data. Except consensus development method and evidence-based method, we introduced text mining technique. Text mining has been widely used in medical research and been introduced into CM research for quickly searching potential information [26–28]. Results derived from text mining reflect the hot point knowledge of relevant diseases which can be considered as a supplement of evidence. Text mining was used for providing more data on forming CM CPG and providing a general basis for avoiding bias generating from unsystematic review or group opinion.

*Secondly*, we chose formal consensus development method instead of informal consensus method. Delphi survey was used to collect consensus from local experts because of its rationality, scientific credibility, and controlled process [29]. To avoid bias generating from individual views, evidence and information derived from text mining process will be used to form questionnaire for leading an effective survey. Participants come from local professional organization to ensure consensus is highly appropriate for local use. Consensus conference was used to provide a preciseness and effective way to achieve consensus on final decision.

*Thirdly*, we synthesize data derived from different resources for supporting CM CPGs.

We set clear criteria and standardized form for synthesizing of data to provide an operable and transparent development process. To facilitate understanding of CM CPG, a standardized reporting guide for CM CPGs is provided as well.

## 4. Conclusion

The outputs of this research will provide CM CPGs for insomnia, chronic gastritis, and cerebral infarction for Hong Kong local use. That is the first CM CPGs for Hong Kong local use and as such is an important milestone. As most of the clinical practices of CM practitioners are still based on the individual experiences, the risk of CM application exists. Therefore, development of CM CPGs to standardize CM clinical practice and ensure CM's secure application is imperative. It will help Hong Kong CM practitioners make decision and enhance efficiency of clinical service to some extent. We initially choose 3 common diseases in Hong Kong according to the suggestion from Hospital Authority of Hong Kong, including insomnia, chronic gastritis, and cerebral infarction with high disease burden and effective practice evidence. If executed successfully, the methods will be used in the future development process of CM CPGs for other common diseases in Hong Kong.

## Figures and Tables

**Table 1:** Matrix table for synthesizing data of CM treatment formula.

Number	CM pattern	Formula	Supported by level of evidence	Support by expert consensus	Frequency in research Literature	Synthesis
1	Pattern 1	Formula 1				Select/reject
2		Formula 2				⋯
*⋮*		*⋮*				*⋮*

**Table 2:** Matrix table for synthesizing data of CM treatment acupuncture.

CM pattern	Acupoint 1	Acupoint 2	Acupoint 3	⋯	Acupoint *N*
Frequency in research literature					
Supported by level of evidence					
Supported by expert consensus					
Synthesis	Select/reject	⋯	⋯	⋯	⋯

**Table 3:** Matrix table for synthesizing data of CM regimen.

Pattern	Item 1	Item 2	Item 3	⋯	Item *N*
Frequency in research literature					
Supported by level of evidence					
Supported by expert consensus					
Synthesis	Select/reject	⋯	⋯	⋯	⋯

**Table 4:** Checklist for reporting CM CPG.

Topic	Specified Items	Description
(1) Title	(1.1) Title (1.2) Subheading	Provide an appropriate title including the specified disease's name using International Classification of Disease (ICD). Relevant CM disease should be noted in subheading.

(2) Introduction	(2.1) Scope (2.2) Development process	Describe goal of the CM CPG with specific details concerning the targeted users, application of regions and countries, and the key clinical questions. A brief description of development process should be Clearfield.

(3) Background	(3.1) Epidemiological details of natural history in WM (3.2) Understanding of disease based on CM theory	Describe epidemiological details (such as incidence, prevalence, and risk factors) and natural history of the relevant diseases in both CM and WM and provide the understanding of disease based on CM theory.

(4) Clinical manifestations	—	Describe the clinical features including patient's history, symptoms, and signs and other relevant information concerning the disease. It is desirable to include both WM and CM perspective.

(5) Diagnostic criteria	(5.1) Current Western medical diagnostic criteria (5.2) CM patterns classification and diagnosis	Describe Western diagnostic criteria and classification of CM pattern including symptoms and signs of each pattern, with the corresponding support data derived from text mining process, survey, and literature evidence.

(6) Intervention	(6.1) Basic principle of intervention (6.2) Herbal medicine treatment (6.3) Acupuncture treatment (6.4) Others options of CM treatments	Provide a specific description of the relevant CM interventions incorporating herbal medicine, acupuncture, or other options of CM treatment. Considering operability of CM CPG, detailed information should be provided like acupuncture manipulation.

(7) Methods	(7.1) Text mining process (7.2) Identified clinical evidence (7.3) Delphi process (7.4) Synthesizing data (7.4) Review and consultation process (7.5) Dissemination (7.6) Implementation (7.7) Updating	Describe the basic information of the key steps in development process, including Delphi process (expert selection, basic information of survey and statistics), text mining process, clinical evidence (search strategy, evaluation of the strengths and quality of evidence, grading of recommendations), synthesizing data process (criteria used in data synthesizing process and synthesizing table), review and consultation process, dissemination, Implementation, and updating plan.

(8) References	—	Provide all the relevant inferences including literature used as evidence.

(9) Appendices	—	Working Group Membership, standardized table evidence and recommendation, and vocabulary.
